# Literature mining of protein-residue associations with graph rules learned through distant supervision

**DOI:** 10.1186/2041-1480-3-S3-S2

**Published:** 2012-10-05

**Authors:** KE Ravikumar, Haibin Liu, Judith D Cohn, Michael E Wall, Karin Verspoor

**Affiliations:** 1 University of Colorado School of Medicine, Aurora, CO 80045, USA; 2 Los Alamos National Laboratory, Los Alamos, NM 87545, USA; 3 National ICT Australia Victoria Research Lab, Melbourne, Australia

## Abstract

**Background:**

We propose a method for automatic extraction of protein-specific residue mentions from the biomedical literature. The method searches text for mentions of amino acids at specific sequence positions and attempts to correctly associate each mention with a protein also named in the text. The methods presented in this work will enable improved protein functional site extraction from articles, ultimately supporting protein function prediction. Our method made use of linguistic patterns for identifying the amino acid residue mentions in text. Further, we applied an automated graph-based method to learn syntactic patterns corresponding to protein-residue pairs mentioned in the text. We finally present an approach to automated construction of relevant training and test data using the distant supervision model.

**Results:**

The performance of the method was assessed by extracting protein-residue relations from a new automatically generated test set of sentences containing high confidence examples found using distant supervision. It achieved a F-measure of 0.84 on automatically created silver corpus and 0.79 on a manually annotated gold data set for this task, outperforming previous methods.

**Conclusions:**

The primary contributions of this work are to (1) demonstrate the effectiveness of distant supervision for automatic creation of training data for protein-residue relation extraction, substantially reducing the effort and time involved in manual annotation of a data set and (2) show that the graph-based relation extraction approach we used generalizes well to the problem of protein-residue association extraction. This work paves the way towards effective extraction of protein functional residues from the literature.

## Background

The rapid pace of genome sequencing adds urgency to efforts for determining the functions of newly sequenced proteins. Analysis of protein sequences and structures can lead to new predictions and discoveries of significant patterns, motifs and functionally important sites. In the context of three-dimensional protein structures, the appearance of certain amino acid residues at key structural positions has a central role in protein function, for instance enabling ligand or substrate binding. For proteins of therapeutic importance, identifying these sites as potential targets is a key early step in drug design.

The biomedical literature is a rich resource for identifying functionally important sites. There is a growing gap between the knowledge embedded in the literature and what has been formalized in genomic databases [1] and we have observed that this is true specifically for functional site information [2]. Efforts to manually catalog functional sites mentioned in the literature are helping but will not fill this gap in the near future, considering the growing pace of the biomedical literature. Hence the overarching goal of our work is to identify such functional sites automatically from the biomedical literature.

We previously addressed this problem by developing automated methods for identifying protein residue mentions in biomedical text, and associating these mentions with functional sites [2,3]. In that work, we showed that detecting residue mentions has some interesting characteristics as a text mining problem: not only do residue mentions exhibit regularities that can facilitate their detection [4], but the independent validation of residue mentions via physical data (the protein sequence) provides an important filtering effect. In this work, we directly take advantage of the available physical information to enable the development of a high-confidence text corpus for training a protein-residue relation extraction system without the need for manual annotation.

Our previous methods [2,3] used curated links from publicly available protein structure records in the Protein Data Bank (PDB) [5] to find relevant PubMed IDs. Residue mentions were extracted from the corresponding PubMed abstracts, and the curated links told us the specific protein that most likely was associated with these residue mentions. In general, however, such curated links between proteins and the literature are limited in number and as such the generalizability of the method is correspondingly limited. When such links to the literature are unavailable, we must instead depend on analyzing the text itself to establish a relationship between a specific protein and a specific residue. Therefore, in this work we explore the application of text mining methods to extract valid protein-residue pairs from abstracts of papers about protein structure. The approach we outline stems from the observation that authors often make statements that explicitly relate a protein to one or more of its constituent residues within the boundary of a single sentence, e.g., "The 162-amino acid PrxV contains Cys residues at positions 73 and 152." (adapted from PMID:10787409). We aim to capture the underlying linguistic structures that express these semantic relationships.

A number of related works have been published with a focus on the extraction of point mutations [6-11]. MEMA [9] and MuteXt [10] use word distance to select among multiple protein-residue pairs extracted out of a text. Mutation-GraB [6] addresses the ambiguity of protein-residue pairs using a weighted graph made up of word bigrams in the text, retaining only the protein-residue pair connected by the shortest path in the graph. MutationMiner [7,8,11] is another notable work that extracts mutations and mutation impact statements from the literature, while also mapping mutation coordinates to protein structures to enable visualization. In addition to extracting mutation mentions from the literature, Witte and Baker [7] attempted to ground the mutated residue mentioned in the text to a specific residue in the protein sequence using regular expressions corresponding to residue motifs detected in the literature. While this strategy of grounding could also be useful in our context, it is less obviously applicable to single residue mentions.

The aims of our study are most closely related to those of Nagel et al., 2009 [4]. That work demonstrated that a predictive model for functional annotation of proteins from the biomedical literature can be derived taking advantage of the information in publicly available resources. In that work, the authors extracted protein-species-residue triples from text using abstract level co-occurrence of the three entities, and validated the triples using reference data in the UniProt Knowledge base [12]. Functional annotation was then addressed with an information-theoretic model that related words in texts to functional categories. Existing UniProt annotations were used to establish both the texts relevant to a protein, and the positive functional annotations of proteins used in training the model.

We focus here solely on developing methods to establish reliable protein-residue associations in situations where validating information may not be available, leaving functional annotation for future work. The Nagel et al. method does not consider any contextual syntactic dependencies between the protein and the residue, an important feature we investigated in this work. We have utilized the gold standard corpus developed by Nagel et al. as an independent test set for the evaluation of our method.

Recently, dependency graphs obtained from full syntactic parsing of text, with their ability to reveal long-range syntactic relations, have been shown to improve biological relation extraction [13-15]. Liu et al. proposed a graph-based approach [16,17] to tackle the event extraction tasks of BioNLP-ST 2009 [18] and BioNLP-ST 2011 [19]. In that method, rules for detecting biological relations are first automatically learned by identifying their key contextual dependencies from full syntactic parsing of annotated texts, captured as a *rule graph*. New relations are then recognized by searching for a subgraph isomorphic to a rule graph within the dependency graphs of complete sentences in the input texts. This approach has also been successfully adopted to extract protein-protein interactions in the biomedical literature [20], demonstrating its generalization capability. In this work, we further explore the potential of this graph-based approach in the novel context of protein-residue association extraction.

## Results

We present the results of our text mining system on both the intermediate step of entity detection and the ultimate task of extraction of protein-residue associations. Each extracted protein-residue relation involves two entities - a protein and a residue. Our representation of a residue annotation includes information about the wild-type amino acid, sequence position, and a possible mutant amino acid type. For example, a relation between the protein "URPTase" and the residue "Arg80" is captured as follows:

Protein: URPTase

Residue:

Wild Type Residue: Arg

Position: 80

Mutant Type Residue: Null

When we evaluate an extracted relation against a gold annotation we consider it to be correct only if every constituent of the extracted relation, i.e., every element of the representation, exactly matches that of the annotation.

Below we present our results of the performance of entity (amino acid residue and mutation) detection on three different data sets; one reviewed by our group and two from external sources. These three resources are annotated manually and therefore we refer to them as the *gold corpora*. In addition, we created a corpus annotated with protein-residue relations using automated methods, which we refer to as the *silver corpus*. We describe these corpora in detail in the Data Sets section.

We did not evaluate the performance of protein name recognition directly on either the gold or the silver corpora. For the gold corpora, we used provided manual annotations of protein names for all downstream processing. In the silver corpus, we retained only those sentences in which the entities detected in the abstracts have been physically validated against a PDB record, as will be described in detail in the Methods, thereby effectively compensating for errors in the protein mention detection.

### Evaluation of entity recognition: amino acids and mutations

We evaluated the performance of amino acid and mutation detection against three gold corpora containing amino acid and/or mutation mention annotations. We first consider performance on a corpus produced by Nagel et al., 2009 [4], summarized in Table 1. We evaluated four different residue or mutation detection systems in total on the Nagel data set. The system (System 1: -SLAA, +SLM; Row 1 in Table 1) which had only single letter mutation (+SLM) patterns but no single letter amino acids, e.g., "H235", (-SLAA) achieved the best performance of 88.92% precision, 98.09% recall and 93.28% F-measure for the extraction of amino acid residue and mutation mentions on the Nagel data set. Nagel et al. reported higher performance (92% precision, 98% recall and 95% F-measure) on the same data set. We also studied the effect of including and excluding the regular expressions for extracting the single letter amino acid residue and mutation mentions on the performance of the residue/mutation detection (Systems 2-4, Table 1). While excluding the single letter mutation patterns significantly affected recall (both System 2 and 3), inclusion of single letter amino acid patterns significantly decreased the precision on the Nagel data set.

**Table 1:** Evaluation of performance of residue and mutation extraction on the Nagel corpus (original annotations)

Evaluation Scheme	Precision (%)	Recall (%)	F-Measure (%)
System1: -SLAA, +SLM	88.92	98.09	93.28
System 2: +SLAA,-SLM	71.86	79.01	75.27
System 3: -SLAA,-SLM	94.78	76.33	84.56
System 4: +SLAA,+SLM	74.42	98.85	84.91
Nagel et al.'s reported numbers	92.00	98.00	95.00

SLAA - Single letter Amino acid patterns; SLM - Single Letter Mutation patterns; + for inclusion of patterns; - for exclusion of patterns

Error analysis revealed that some of the errors in residue/mutation detection are due to the difference in the notion of what we consider a valid residue mention as compared to the annotation of the Nagel corpus. For example, while the Nagel corpus annotation considers "His43-Asp88-Ser182" to be a single residue, our system detects them as three individual residue mentions. This results in three precision errors and one recall error. In order to bring the annotation in line with our guidelines, we re-annotated such instances in the Nagel corpus and re-evaluated them using the modified annotation set ("Modified Nagel"). A significant increase in performance demonstrates the impact of this modification (Precision: 91.64%, Recall: 98.50%; F-measure: 94.95%). As summarized in Table 2, the new results are competitive with the figures reported by Nagel et al., 2009 [4].

**Table 2:** Evaluation of performance of residue and mutation extraction on the Nagel corpus (Modified per our annotation guidelines)

Evaluation Scheme	Precision (%)	Recall (%)	F-Measure (%)
System1: -SLAA, +SLM	91.64	98.50	94.95
System 2: +SLAA,-SLM	74.38	79.17	76.70
System 3: -SLAA,-SLM	96.23	77.27	85.71
System 4: +SLAA,+SLM	77.41	98.88	86.84

SLAA - Single letter Amino acid patterns; SLM - Single Letter Mutation patterns; + for inclusion of patterns; - for exclusion of patterns

Table 3 summarizes our performance on the task of mutation extraction against the Mutation Finder [21] corpus. We achieved a performance comparable to their reported evaluation on their development (Precision : 96.81%; Recall: 82.9% ; F-measure : 89.32%) and test corpora (Precision: 95.61%; Recall: 81.59%; F-measure: 88.04%). Caporaso et al. 2007 [21] reported a precision of 98.40%, recall of 81.90% and F-measure of 89.40% on their test corpus. Although the MutationFinder system (http://mutationfinder.sourceforge.net/) outperforms our system on this data set, it does not meet our requirement to recognize individual amino acids and so we cannot substitute their system for ours.

**Table 3:** Table 3

a. Evaluation of performance of mutation extraction on MutationFinder corpus				
**Corpus**	**System**	**Precision (%)**	**Recall (%)**	**F-Measure (%)**

Development	Our system	96.82	82.91	89.32

Test	Our system	95.61	81.59	88.04
	
	MutationFinder	98.40	81.90	89.40

b. Evaluation of performance of residue and mutation extraction on LEAP-FS corpus				

**Corpus**		**Precision (%)**	**Recall (%)**	**F-Measure (%)**

LEAP-FS		85.23	87.93	86.56

To assess how well our patterns that identify residues and mutations generalize to full text articles, we evaluated the performance against a new gold standard set of 50 full-text articles, which we dub the LEAP-FS corpus for the system it was built to support [2]. This corpus was manually annotated according to our guidelines. The system achieved a precision, recall, and F-measure of 85.23%, 87.93% and 86.56% respectively. An appreciable drop in the precision in identifying the amino acid residues and the mutations in the LEAP-FS corpus is predominantly due to the inclusion of patterns to capture single letter amino acid residues as seen in Table 3. As noted in the Data Set section of this paper 16% of the amino acid residue annotations in the LEAP-FS corpus include single letter amino acid abbreviations. If we do not include patterns to extract single letter amino acids, the system would have low recall. Another source for precision errors is due to the identification of residues or mutations mentioned in non-primary sections of the paper (e.g., Title and References sections). Correct treatment of information in such sections is pointed out by Cohen et al. 2010 [22] to be a challenge in processing full-text articles.

Some of the errors that were common, irrespective of the document type (abstract or journal article), can be attributed to regular expressions that were intended to capture single letter point mutations such as "S235A", but instead misidentified gene names such as "A8R", "A23R" and cell lines such as "R2C" as mutations. Including the regular expressions for extracting both single letter amino acid residues and single letter mutations resulted in very low precision (74%) while recall was 99% and F-measure 84%. Disabling these regular expressions increased precision (95%) with a sharp decline in recall (76%) and an overall drop in F-measure to 85% which shows that single letter mutation mentions contribute significantly to recall. We continue to work to refine our entity recognition strategies.

### Evaluation of protein-residue relation extraction

Our relation extraction methods are described in detail in the Methods section. In brief, we employed a system that learns rules, in terms of syntactic patterns, for extracting specific relations. The method requires appropriately structured training data. Since we reserve the small number of examples of protein-residue relations in the Nagel corpus for testing, we pursued a method for automatically constructing reliable training, development, and test data. Again, the construction of this so-called "silver" corpus is described in detail in the Methods.

The silver corpus consists of sentences that contain at least one protein mention and either an amino acid or mutation with a physically validated relationship to the mentioned protein. During training, both the protein and the validated associated residue mentions are made available to the pattern learning algorithm. During testing, only the protein mentions are pre-identified. The relation detection method we used addresses extraction of the protein-residue associations from individual sentences and therefore only relations expressed within a given sentence are targeted.

We were able to automatically build rules that capture the underlying syntactic relationships for 1,741 physically validated high confidence protein residue relations. After removing duplicate rules, we obtained 1,311 rules to relate proteins and residues in the text. We utilized a performance-based rule ranking method to evaluate each rule. We then matched each rule to sentences in the development set using the subgraph matching approach proposed in [16]. Rules that produce at least one relation prediction were further ranked by PRC(r_i_), the precision of each rule r_i_, computed via Equation 1.

(1) $$\text{PRC(}{\text{r}}_{\text{i}}\text{) = }\frac{\#\;\text{Correctly}\;\text{predicted}\;\text{associations}\;\text{by}\;{\text{r}}_{\text{i}}}{\#\;\text{predicted}\;\text{associations}\;\text{by}\;{\text{r}}_{\text{i}}}$$

Based on a previous investigation [17], rules with higher PRC(r_i_) values tend to produce fewer false positives. We therefore retained the rules with a PRC(r_i_) higher than 0.25. For rules that do not make any predictions on the development data, we retain them in the hope that they may contribute to the relation extraction from the testing data. Without affecting the recall much, this process helped to improve the precision of the relations extracted from the development data.

Table 4 shows the results of the protein-residue relation extraction on the development and test portions of the silver corpus based on two matching criteria, a stricter criterion E+P+A, and a relaxed criterion E+P*+A*. The stricter matching criterion (E+P+A) requires that edge directions and labels of all edges (E) be identical, all tokens (A) and their associated POS tags (P) be identical for the edges and the nodes of a rule and a sentence to match with each other. The relaxed criterion (E+P*+A*) requires only that the lemmatized form of tokens and the relaxed POS tags be same. The relaxation of POS tags means that for nouns, the plural form is allowed to match with the singular form, and proper nouns are allowed to match with regular nouns; for verbs, past tense, present tense and base present form are allowed to match with each other. The drop in the precision offsets the marginal gain in the recall due to this relaxation of the matching criteria, leading to a small increase in the overall F measure.

**Table 4:** Evaluation of subgraph matching and co-occurrence baseline approach for protein-residue relation extraction on silver corpus.

Corpus	Corpus	Precision (%)	Recall (%)	F-Mes (%)
Development Corpus	E+P+A	80.26	77.05	78.62
	E+P*+A*	79.10	78.10	78.60
	E+P+A+Rule ranking	81.20	76.42	78.74
	E+P*+A*+Rule ranking	79.35	77.68	78.51
	Sentence co-occurrence baseline	59.45	100	75.28

Test Corpus	E+P+A	84.07	79.43	81.69
	E+P*+A*	82.72	80.10	81.39
	E+P+A+Rule ranking	86.83	78.26	82.32
	E+P*+A*+Rule ranking	83.60	78.43	80.93
	Sentence co-occurrence baseline	62.42	100	76.86
	Approximate subgraph matching (ASM) with distance threshold 0.6	81.96	86.62	84.22

E+P+A - Match edge labels, Parts of speech, All tokens; E+P+A* - Match only Edge labels and Parts of speech.

We further implemented two simple co-occurrence-based methods to serve as baselines to compare with the graph-based approach. The methods extract all possible protein-residue relations, in the first method from within each training sentence and in the second method from a complete PubMed abstract. Where multiple protein or residue mentions occur within a single sentence or abstract, all pairwise combinations were extracted. Table 4 also lists the results for this sentence-level co-occurrence baseline on the silver corpus.

We observe that although the precision achieved by the graph-based approach significantly outperforms the baseline method, about 20% of the protein-residue associations are missed. We attribute this to the fact that these relations are described in grammatical structures that are not covered by the existing rules induced from the training sentences. This lack of coverage can be attributed to two factors: to the presence of novel syntactic structures in the test set that were unseen in the training set, or to the relation being expressed in a syntactic construct that the method cannot capture. For the latter case, such constructs tend to be complex, involving a long dependency path from the protein to its associated residue in the sentence. Relations that consist of these structures are not recognized, as no matched rules will be returned under the framework of the current exact subgraph matching.

In order to further explore the generalization potential of extracted rules, we additionally applied an approximate subgraph matching (ASM) algorithm proposed in [23] to this relation exaction problem for comparison to the exact subgraph matching method. This penalty-based approximate subgraph matching measures the distance between dependency graphs of a rule and a sentence by the weighted summation of three components: subgraph strutural distance, dependency label distance and dependency directionality distance. Since the algorithm respects the elements of a rule, and allows only variations in the sentence graph, e.g., nodes or edges of the sentence graph to be skipped with penalty, the matching process corresponds to a search for a subgraph within the sentence graph that is approximately isomorphic to the rule graph. A distance threshold is used in the algorithm to regulate the relation extraction performance. Compared to the restrictive exact subgraph matching approach, the ASM allows partial matching by giving the corresponding penalty and using distance threshold to determine the degree of similarity between an event rule graph and a sentence graph. The protein-residue associations extracted by this extended algorithm naturally subsume the results from the exact subgraph matching.

The standard experimental setting for performing the ASM algorithm is to learn and optimize the distance threshold from the training or development data, and then apply the resulting threshold to the test set and report the relation extraction performance. In this work, however, the application of ASM is a proof-of-concept experiment to demonstrate that the algorithm is capable of retrieving more potential protein-residue associations encoded by longer-range dependencies and various syntactic relationships in sentences, which cannot be captured by the exact matching approach, while still maintaining the extraction precision at the high level.

Therefore, we conducted this approximate matching experiment only on the test portion of the silver corpus, and investigated the effect of tuning the distance threshold to the overall performance of the protein-residue association task on this data set. While this does mean that the results we present must overestimate the performance that would be achieved on unseen text, it allows a direct comparison to the test results of the exact subgraph matching method.

**Figure 1:**
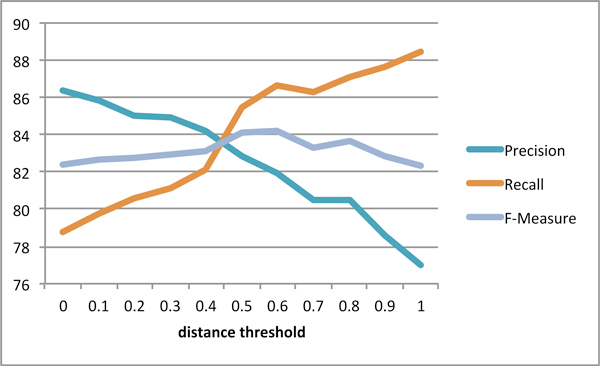
**Effect of distance threshold on the performance of protein-residue relation extraction on the test portion of silver corpus in approximate subgraph matching**.

We have experimented with a series of distance thresholds ranging from 0 to 1 with an interval of 0.2. While the threshold 0 corresponds to the exact subgraph matching with the matching criteria "E+P*+A*+Rule Ranking" (row 4 in the Test corpus section of Table 4), the distance threshold 1.0 represents the maximal degree of approximate matching allowed for this work. The results shown in Figure 1 illustrate that the F-measure is the highest at the threshold 0.6, and the precision drops significantly when a bigger threshold 1.0 is used. The approximate matching algorithm was able to increase the recall by 6.5% over the best recall on the Test corpus achieved with exact subgraph matching, shown in Table 4, while still retaining the precision at the 82% level, leading to a significant 3% increase in F measure.

Table 5 shows three different evaluations against the hand-annotated gold set of Nagel et al. 2009 [4]; the two co-occurrence baselines and the exact subgraph matching approach. For evaluating the performance on extracting protein-residue relations on this data set, we considered only the 197 protein-residue relations involving a residue at a specfic position, ignoring bare residue mentions, in accordance with our residue annotation guidelines.

**Table 5:** Evaluation of protein residue relation extraction on Nagel corpus

Method	Precision (%)	Recall (%)	F-Measure (%)
Abstract level co-occurence	57.10	100	72.69
Sentence level co-occurence	63.50	84.77	72.61
Subgraph matching	85.09	69.54	76.54
Subgraph matching (Modifed annotations)	90.38	71.57	79.89

## Discussion

### Pattern learning

The performance of the dependency graph-based approach is consistent on both the silver and gold corpora. The strictest matching criteria achieved a good performance on both the development and test sets. While relaxing the token matching constraints did not have significant impact on the overall F-measure, trading increased recall for lower precision, the performance-based rule ranking contributed to a marginal increase in the F-measure. The rule ranking significantly boosted precision, but sacrificed some recall. Furthermore, without sacrificing precision, the approximate subgraph matching approach significantly improved recall, showing an encouraging potential for relation extraction applications.

The graph-based approach to protein-residue relation extraction considerably outperformed the sentence-level co-occurrence baseline on both the development and test set. While the sentence based co-occurrence method achieved 100% recall, as is expected based on how the silver corpus was constructed (using only sentences with an overt protein and amino acid mention), its precision was under 60%. In contrast, the exact subgraph matching approach has a more balanced performance, achieving a highest precision of 86% and a highest recall of 78%.

The results in Table 4 provide evidence of one interesting property of our graph rule-based method, through the increased performance on the test set as compared to the development set. While evaluating the development corpus, only event rules derived from the training data were included in the rule set. While evaluating the test corpus, event rules derived from both training and development data were included in the rule set. Unlike traditional machine learning-based methods in which parameters are heavily tuned to the development corpus, the graph rule-based method shows an advantage when using the distant supervision framework, as rules/patterns learned from co-mentions of pairs of entities known to interact are not prone to over-fitting to a training corpus. Similar to the performance reported in [16,17], therefore, the method is more generalizable across different datasets, leading to a comparable performance on the test corpus to that of the development corpus. The higher association extraction performance on the test corpus indicates a difference in the underlying data distribution between the two corpora, also supported by the somewhat increased performance of the co-occurrence baseline on the test data as compared to the development data, as well as the potential contribution of event rules induced from the development corpus.

### Pattern learning: error analysis

The higher precision of the graph-based method indicates that there was substantial ambiguity within the sentences of the corpus that can be resolved using syntactic relations. The lower precision of the baseline method derives from it postulating relations among a protein and an amino acid that do not correspond to a physically valid association. It can be inferred from our results that physically valid protein-residue relations are expressed in syntactic constructs that clearly relate the two constituent parts. For instance, consider the sentence "In previous studies we found that the primary reason for selectivity is that *Asp597 *in *nNOS*, which is *Asn368 *in *eNOS*, provides greater electrostatic stabilization in the inhibitor complex." (PMID 16285725). The co-occurrence method would identify 4 relations, *nNOS-Asp597, eNOS-Asn368, nNOS-Asn368*, and *eNOS-Asp597*. Only the first two of these are correct, leading to two false positives. The graph-based approach, in contrast, only identifies the 2 correct relations, based on a rule capturing the pattern "*Residue *in *Protein*".

Error analysis further revealed that a long-distance path between the protein and residue mentions, where the relationship description extends beyond clausal boundaries, is one of the major factors contributing to the lower recall of the graph-based method. Consider the following sentence "The crystal structure of recombinant *ag85C *from M. tuberculosis, refined to a resolution of 1.5 A, reveals an alpha/beta-hydrolase polypeptide fold, and a catalytic triad formed by *Ser 124*, *Glu 228 *and *His 260*". While the sentence or abstract level co-occurrence would capture three protein-residue relation pairs (ag85C-Ser124, ag85C-Glu228, and ag85C-His260) from the sentence, there is no rule that captures such long-distance relations, leading to recall errors. However, this recall problem can be significantly ameliorated with approximate subgraph matching, as we have demonstrated.

Relaxing the criteria of strict match for all tokens in the rule contributed to a drop in precision on the test set from 84% to 82% (E+P+A vs. E+P*+A*) and from 86% to 83% in the rule ranking scenario (E+P+A+Rule Ranking vs. E+P*+A*+Rule Ranking), as shown in Table 4. Errors due to syntactic parsing may also contribute to precision errors.

### Discussion of results on the gold corpus

Our evaluation results on the independently annotated Nagel gold corpus (Table 5) show that although the exact subgraph matching approach achieved a high precision - similar to that on the silver corpus - it had a lower recall than on the silver corpus. While the recall of the co-occurrence baseline methods on the gold standard is high at both the abstract (100%) and sentence (76%) level, the precision of both baseline approaches was very low, resulting in a lower overall F-score than the sub-graph matching results. The approximate matching approach achieved not only a higher recall but retained a very high precision.

In the Nagel gold corpus, there were a significant number (18%) of protein-residue relations that span multiple sentences, placing an upper bound on the recall of any sentence-bound method and therefore contributing to the lower recall of our sentence-based approach. The impact of this can be seen clearly by comparing the recall of the abstract co-occurrence baseline with the recall of the sentence co-occurrence baseline in Table 5. Examination of these cases, found in 11 abstracts, indicates that often the relevant protein is introduced early on in the abstract and implicitly referenced in subsequent sentences. There are in some of these cases multiple intervening sentences between the protein mention and the residue mention. For instance, in PMID 7608980, the protein *abrin-a *is introduced in the first sentence of the abstract, while its residue, *Tyr74*, is discussed in the sixth sentence. This indicates that a simple window-based constraint on abstract co-occurrence would be inadequate to improve recall without also impacting precision. Quite sophisticated coreference resolution would be required to handle these cases; for instance, in the sentence "The positions of invariant active site residues remain the same, except the position of Tyr74" there is no explicit reference to a protein, even indirectly (e.g., via a pronoun such as "it", or via a reference such as "the protein").

Error analysis of results on the gold data set revealed certain errors in mutation detection due to partial extraction of the wild type residues with positions while missing the mutant residue. For example, consider the phrase "Mutation of Tyr-196 in glycogenin-2 to a Phe residue abolished ..." (PMID 9857012) in which we extract Tyr-196 as amino acid and fail to detect the Tyr196-Phe mutation. Hence we extract only the wild-type residue-protein pair (Tyr196/glycogenin-2), and fail to detect the wildtype residue with mutant and protein pair (Tyr196-Phe/glycogenin-2) pair. This could arguably be considered correct for our purposes as the protein-wild-type residue relational pair is the one which is biologically significant, but it does not exactly match the annotation.

There are other similar errors. For example from the phrase "Both ATP binding [Vps4p-(K179A)] ..." (Nagel corpus, PMID 12953057), we extracted a relation between the protein "Vps4p" and "K179A" which seemed to be correct. On manual inspection we found that errors were due to the mismatch in the residue slot. The Nagel annotation for the protein-residue relation specifies "Vps4p" to be the protein and the full phrase "Vps4p-(K179A)" in the residue slot. On the other hand, our extraction fills the residue slot with the mutation information alone ("K179A"). This annotation is in fact not even consistent with the annotation of most other protein-residue relations in the Nagel corpus. If we correct for these mismatches, precision, recall and F-measure increase to 90.38%, 71.57% and 79.88%, respectively. Our overall performance indicates that the subgraph pattern learning approach with very high precision and good recall is more reliable than the co-occurrence baseline approaches, and is suitable for this information extraction task.

Our approach generates some false positives in which the relationship extracted involves the incorrect protein when there is more than one protein mentioned in the sentence. For instance, the correct relations for this sentence from PMID 11108838, "Using a functional assay based on inhibition of leptin mediated reporter induction, and using phosphopeptide affinity chromatography we show binding of *SOCS3 *to the highly conserved phosphorylated *Tyr-985 *and *Tyr-1077 *motifs within the mouse *leptin *receptor" are between leptin and each tyrosine. Our system instead proposes a relation between SOCS3 and each tyrosine. This sentence proved difficult for the parser; the prepositional phrase "within the mouse leptin receptor" was attached too high, to the verb "using" and as such the correct relation would have been difficult to identify via the syntactic analysis. Hence the example also represents two false negatives for our method.

We have identified some omissions in the Nagel et al. annotation. For instance, in the sentence, "Our results indicated that human LAB was primarily phosphorylated on three membrane-distal tyrosines, Tyr(136), Tyr(193), and Tyr(233)" from PMID 14722116, the relations between LAB and the three residues in that sentence appear to be valid protein-residue pairs, but are not annotated by Nagel et al. Rather the gold annotation includes only a relation from LAB to a more generic phrase "Tyr to Phe" from another sentence in the abstract. As we have not exhaustively reviewed the Nagel et al. annotation we used as our gold, we cannot determine the full impact of such errors on the performance of the various systems we tested.

### Silver corpus construction

A notable contribution of our approach is to take advantage of the information in the existing repositories through distant supervision to create positive training instances for the pattern learning system. Although we are able to create high confidence protein relationship statements without manual effort, our approach has some limitations. There is a potential loss of true positive relationship statements, since (a) relationships may not be expressed exclusively within a single sentence, but rather using co-reference or simply by establishing a focus protein for the paper as a whole, and (b) the physical validation we utilize may miss some valid relationships due to variations in sequences, e.g., numbering differences in the PDB. This will result in loss of potential training patterns, which may in turn cause a decrease in recall.

## Conclusions

Through this work we have demonstrated that the application of a subgraph matching-based relation extraction approach generalizes well to the problem of extracting protein-residue associations. It achieves much better performance than baseline co-occurrence methods. The task itself has broader significance for protein function prediction and subsequent drug discovery, given the context of our ongoing research of into integrating evidence extracted from the biomedical literature into a protein function prediction system [2,3].

Furthermore, we have shown that the creation of an annotated data set through distant supervision is highly effective for quickly building high quality training instances. The patterns induced from such training data not only achieve high performance on the automatically created test data but also perform well on an independent, manually annotated gold corpus. The results encourage us to explore the use of distant supervision for other information extraction tasks in biology of higher complexity. Automatic creation of training data as shown in this study will significantly reduce the manual effort in creating gold corpora without much compromise on the overall performance of information extraction.

## Data sets

We used multiple manually annotated data sets (Nagel, MutationFinder and LEAP-FS corpora) for evaluating the performance of our entity detection and protein-residue relations in addition to the silver corpus we constructed. While we introduce the statistics of the silver corpus in Table 6, we briefly review the Nagel corpus, the Mutation Finder corpus and the LEAP-FS corpus consisting of annotations over full-text articles. All three of the corpora are available at http://bionlp-corpora.sourceforge.net/proteinresidue/. We used the version 0.2 of our corpora for the current study.

**Table 6:** Protein residue relation statistics of silver corpus

Parameter	Number
Total number of abstracts	18,045
Total number of sentences	138,790
Total sentences with protein names	41,722
Total sentences with at least one amino acid or mutation	13,729
Sentences with co-mentions of protein-amino acid (or) mutation	5,256
Sentences with validated protein-residue relations	2,516
Physically validated protein-residue relations	2,814
Total abstracts with validated protein-residue relation	1,728

### Nagel corpus

Nagel et al. 2009 [4] built a corpus of 100 PubMed abstracts with annotations for protein residues and mutations. It also includes annotations for organism-protein-residue triplet annotations. The corpus has 262 amino acid residue/mutation annotations and 232 protein-residue relations in total. Among those 232 annotations, 35 of the residue mentions were not site-specific, that is they did not include a specific position/location for the residue. This left 197 relations involving a protein mention and position-located residue mention. These 197 pairs were considered as the final gold standard set of relation pairs in our evaluation on this corpus.

### MutationFinder corpus

Caporaso et al. 2007 [21] created two independent gold standard data sets: one for developing their patterns to extract mutations (Development corpus) and the other to evaluate their performance of mutation extraction (Test corpus). Their development corpus consists of 305 abstracts with 605 point mutation mentions and their test corpus consists of 508 abstracts with 910 mutation mentions.

### LEAP-FS corpus

While the silver corpus and the Nagel corpus utilize PubMed abstracts, we manually annotated 50 full text articles for amino acid residues and mutation mentions in them which we call LEAP-FS corpus. These full text articles were selected from among 18,045 PubMed IDs derived from the primary references of the PDB entries used in the LEAP-FS experiments [2] and described in detail in the "Collection of PubMed abstracts" in the Methods section. Annotation of the LEAP-FS corpus (50 full text articles) was performed in Knowtator [24], a plugin for the Protégé framework [25]. While the Protégé framework supports defining an ontology for the annotation, the Knowtator plugin supports the association of annotation classes in the ontology to text sources. Our ontology for annotating the amino acid residue and mutations was simple and consists of three main classes: Amino Acid Residue, Mutation and Sequence. The amino acid residue class consists of two slots, one for the residue, and the other for the position. The amino acid slot is always normalized to three letter amino acid code. For instance, a textual occurrence of an amino acid residue "Histidine-129" will annotated as "His" in the amino acid residue slot, while the "129" will be filled in the position slot. The mutation class consists of three major slots: wild type residue, mutant residue and the position of the site. For a mutation "S230G" occurrence in the text the wild type slot will be filled as "Ser", the mutant residue slot will be filled as "Gly" and the position slot as "230". The start and the end position of the text span pertaining to each annotation is recorded so that we can easily traceback the location of the annotation in the text.

The LEAP-FS corpus contains 3120 annotations in total, out of which 2831 were amino acid residues and 289 were mutations. A notable aspect of this corpus is that out of the 2831 amino acid annotations, the three letter amino acid residue mentions (e.g., His-161 (or) Asp280) and single letter amino acid residue mentions (e.g., D450) constitute 80% and 16% of the residue mentions, respectively, while the full amino acid residue names (e.g., arginine-21) and linguistic expressions (proline at position 127) constitute only the remaining 4%. The corpus has been annotated by a single annotator but is currently being annotated by a second annotator to support eventual calculation of inter-anntator agreement.

## Methods

### System architecture

**Figure 2:**
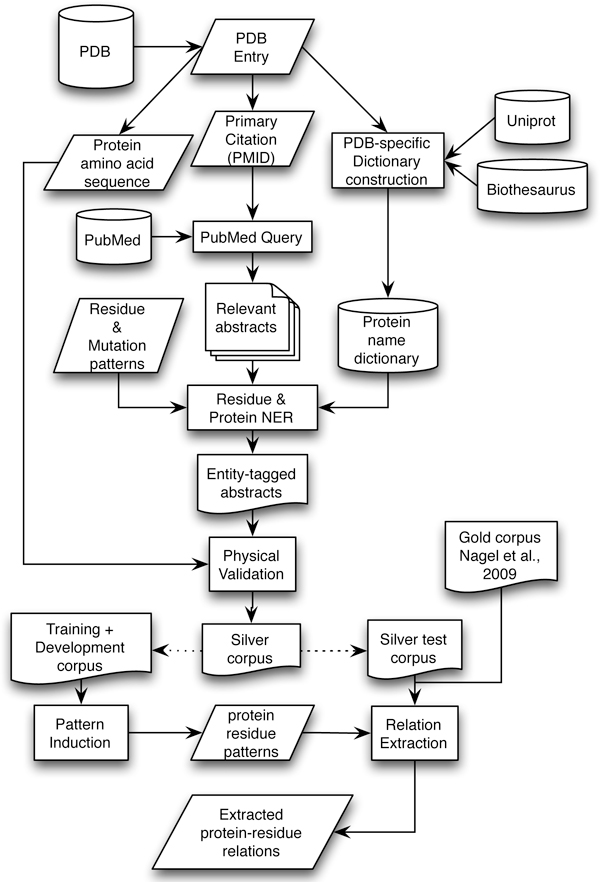
**System Architecture**.

Figure 2 illustrates the overall architecture of our approach to building an information extraction system for protein-residue relations. The pipeline starts with the collection of primary references from the PDB (18,045 abstracts). Each abstract is split into sentences and then protein names, amino acid residues and mutations are recognized and annotated. All possible protein-residue pairs occurring within each sentence are physically validated. The abstracts containing sentences with physically validated relations form the silver corpus (1728 abstracts). These abstracts are divided into three sets - training, development and test corpora. The dependency representation of all abstracts in the silver corpus are obtained using the Stanford Parser [26]. The syntactic patterns to extract the protein-residue association are induced from the training corpus and further refined against the development set. The rules from the training and the development set are run against the test portion of the silver corpus and evaluated for performance of protein-residue relation extraction. The rules are also evaluated against an independent, manually annotated gold corpus. More details are provided below.

The premise of our work is that there is a set of frequently occurring rules, or linguistic patterns, that match a majority of protein-residue relations. We suggest that a rule characterizes the typical contextual structure of the relation. As described, we explored a graph-based approach [16,17] based on the syntactic dependency parse graph of annotated sentences to automatically learn linguistic rules for extracting protein-residue pair relations from abstracts.

### Silver corpus construction

We created a "silver standard" corpus that contains high-confidence protein-residue relationships, substantiated by a physical match of that specific residue to that specific protein. We use this silver corpus for both inferring our rules to extract protein-residue association and testing our extraction accuracy. This strategy for creating annotated data uses the distant learning paradigm where external knowledge drives relation extraction learning [27,28]. Biological knowledge bases have been shown to be effective sources of knowledge for weakly supervised information extraction methods [29,30], and our work provides additional confirmation of the effectiveness of this approach. Here, we use the PDB as our external biological knowledge source.

To create the silver corpus, we acquired PubMed citations for each PDB entry. A dictionary of protein names was compiled from the BioThesaurus database [31] for all entries in the PDB. Amino acid and mutations were tagged in the PubMed abstracts using regular expressions while the protein names were identified through dictionary look-up [32] . In the following sections we describe our methodology for construction of the silver corpus in more detail.

### Collection of PubMed abstracts

To enable the acquisition of the linguistic constructs corresponding to expressions of a protein-residue relationship, we first compiled a corpus of relevant abstracts from MEDLINE. We started with a set of 37,980 PDB entries linked to the 106,411 SCOP domains in the dataset we used in our prior work [2,33,34]. To obtain relevant abstracts, we extracted PubMed IDs for the primary references from the PDB entries. As described in [2], the final corpus consisted of 18,045 abstracts representing the primary references for 30,816 PDB entries. Due to the use of the PDB-PMID relation, each abstract in our corpus was therefore known to be relevant to a specific protein.

### Pre-processing

All abstract text in the corpus was split into sentences using the LingPipe sentence detector [35], tokenized using PennBioTokenizer and annotated with part of speech tags using the GENIA tagger [36].

### Detection of amino acids, mutation and protein names in the text

Our approach to identify residue mentions and mutations in abstracts and full text articles is similar to earlier work [4,7-9,11,21,37], with additional patterns to handle other linguistic variations. Table 7 provides the details of our pattern definitions and some of the regular expressions along with the examples. These patterns are designed to identify both the amino acid and the particular position where it occurs in the protein sequence. For example the pattern "His0[1-9]+" would match "His154" in the text which corresponds to an histidine residue at position 154 in the protein sequence. Our ability to extract point mutations from the text ranges from simple regular expressions such as "S232A", "Cys265Arg", and "Ser-37→ Ala", "Ser59-Histidine" to linguistically enriched expressions such as "serine 32 mutated to alanine", "serines at positions 32 and 73", and "mutation of cysteine 467 in p53 to ala". Our patterns handle Unicode characters used in such mentions, particularly in full text articles.

**Table 7:** Pattern definitions and regular expressions to detect amino acid residues and mutations in the text

Pattern name	Pattern Meaning	Expressions
RES-S	Single letter amino acid code	[ARNDCQEGHILKMFPSTWYVOUBZX]
RES-T	Three letter amino acid code	([aA]la|ALA|[aA]rg|ARG| [aA]sn|ASN|[aA]sp|ASP| [cC]y|CYS|[gG]ln|GLN| [gG]lu|GLU|[gG]ly|GLY| [hH]is|HIS|[iI]le|ILE| [lL]eu|LEU|[lL]ys|LYS| [mM]et|MET|[pP]he|PHE| [pP]ro|PRO|[sS]er|SER| [tT]hr|THR|[tT]rp|TRP| [tT]yr|TYR|[vV]al|VAL| [pP]yl|PYL|[sS]ec|SEC)
RES-F	Full amino acid names	([aA]lanine|[aA]rginine| [aA]sparagine| [aA]spart(ate|ic acid)| [cC]ysteine|[gG]lutamine| [gG]lutam(ate|ic acid)| [gG]lycine|[hH]istidine| [iI]soleucine|[lL]eucine| [lL]ysine|[mM]ethionine| [pP]henylalanine|[pP]roline| [sS]erine|[tT]hreonine| [tT]ryptophan|[tT]yrosine| [vV]aline|[pP]yrrolysine| [aA]spartic acid |[aA]sparagine|[gG]lutamic acid|[gG]lutamine)
POS	Residue Position	0[1-9]{1,5}
WTRES	Wild type residue	(RES-S|RES-T|RES-F)
MUTRES	Mutant residue	(RES-S|RES-T|RES-F)
UNIARR	Unicode character for arrows	\\u2192,\\u21D2
UNIDASH	Unicode character for dash	\\u2013
GRAMMAR	Grammatical expressions	residues? at positions?|for| position|residues? (in|on|at) |substitutions? at|always exists as|at positions?|mutated to|substituted by
POSCOORD	Co-ordination of residue position	POS(,\\s?POS)* (and|or) POS [e.g., 75, 76 and 78, 82 and 95]
AMINOCOORD	Co-ordination of amino acid residues	(RES-T|RES-F)(,\\s?RES-T|RES-F)* (and|or) (RES-T|RES-F) [e.g., Alanine and Valine]
WORD	ANY WORD	
PREP	Prepositions	in, at, on, within, of

Pattern names are shown in THIS FONT and can be themselves used within other regular expressions.

**Figure 3:**
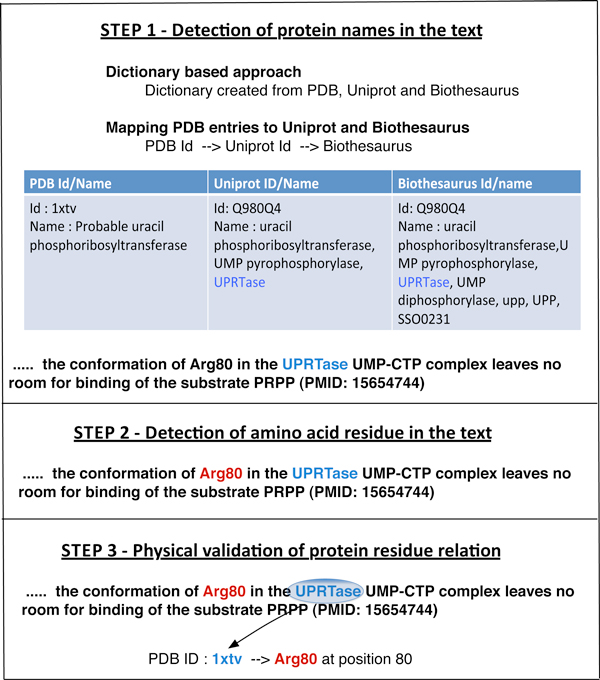
**Physical validation of protein residue relation**.

We used dictionary lookup with fuzzy matching [32] to recognize protein names in the abstracts. The dictionary of protein names was compiled from the BioThesaurus database [31] a system designed to map a comprehensive collection of protein and gene names to UniProt Knowledgebase (UniProtKB) [12] protein entries. Uniprot accession number is used as an intermediate link to map the PDB [5] entries to the one in the BioThesaurus. Step 1 shown in Figure 3 illustrates the details of how the protein name that occurs in the text are detected.

### Physical validation of text residues

Figure 3 illustrates the details of the physical validation of amino acid residues or mutations extracted out of the text. The process of validating residues mentioned in the text to the PDB database, which we refer to as physical validation, comprises the following steps, repeated here from [2] for completeness.

• The text occurrences for each abstract are grouped by site number to determine if amino acid mentions for that site are consistent - i.e., there is a single primary and, where present, a single mutation amino acid for the site.

• A list of text residues was generated by mapping each consistent site in Step 1 to PDB entries using the PubMed ID of the primary reference.

• The text residues are matched against physical residues in a corresponding PDB entry. This is done by exactly matching the text residue site number with a PDB residue number and then either the wild type or mutated text residue amino acid name with the PDB amino acid. If a text residue matches residues in more than one mmCIF entity in a PDB entry, it is labeled as an ambiguous match. Text residues with an unambiguous match are retained, while residues that do not match or have an ambiguous match are eliminated from further analysis.

The requirement of a physical match between a residue mentioned in an abstract and a residue of an abstract-associated protein has the effect of filtering out extraneous residue mentions; we effectively filter out false positives of the text extraction method if the text occurrences cannot be linked to any physical residue.

### Selection of sentences containing protein-residue relations

To facilitate both training and testing of our method, we require annotated examples of high confidence protein-residue relationships. In the current work our focus is only on extracting the protein-residue pairs that occur within a single sentence.

To enable the creation of a set of sentences that contains high-confidence relationship statements, we selected all sentences containing both a protein name in our PDB-specific protein name dictionary, as well as an amino acid mention as our initial pool of sentences. We further filtered this initial pool to only those sentences containing a physically validated relationship: i.e., sentences that contain a protein-residue co-occurrence substantiated by a physical match of that specific residue to the mentioned protein, and where the sentence comes from an abstract explicitly associated with that protein in its PDB record. Consider the following sentence from the PMID: 15654744 "CTP binding affects the conformation of Arg80, and the *Arg80 *conformation in the *UPRTase*-UMP-CTP complex leaves no room for binding of the substrate PRPP." The protein name dictionary look up detects "*UPRTase*" as protein and the regular expression detects *Arg80 *as the residue. This protein-residue pair relation is validated via the PDB entry "**1xtv**", with PMID 15654744 given as the primary citation. The data in Table 6 show that a substantial number of co-localized protein-residue pairs were filtered in the physical validation step.

### Preparation of data set

The 1,728 abstracts (last row in Table 6) that contained physically validated high confidence protein-residue relationships were randomly divided into training, development and test sub-corpora using a random number generator. While 80% of the abstracts were used for training (1106 abstracts) and development (276 abstracts) the remaining 20% were reserved for testing (346 abstracts). The silver corpus had 2814 (last but one row in Table 6) physically validated relations out of which the training set and the development set contain 1,741 and 475 physically validated relationships respectively, the test set contains 598 physically validated protein-amino acid/mutation relations. The file format of all the data sets in the silver corpus was prepared as per the guidelines defined in the BioNLP shared task 2011 [38]. In our current work each protein-residue relation is treated as an event to be consistent with the shared task.

### Extraction of protein-residue relations

We learn the protein-residue relation rules from the silver corpus labeled training sentences (972 abstracts) using a graph-based rule induction method [16,17]. We briefly describe the algorithm here; for more details see [16,17]. We start with the dependency graph produced by the Stanford parser [26,39] for each training sentence, which captures the syntactic dependency relations among all words in the sentence. Edge directions are removed, transforming the directed graph into an undirected graph, where a path must exist between any two nodes since the graph is always connected. For each relation in the training set, the shortest dependency path in the undirected graph connecting the protein to the amino acid or mutation node is selected. The union of all shortest dependency paths is then computed, and the original directed dependency representation of the path union is retrieved and used as the graph representation of the event. Through this process, each gold event is transformed into the form of a biological event rule, which contains the event type, event participants and the corresponding graph representation.

The shortest path between the two entities (protein and amino acid/mutation) is often assumed to contain the most valuable information about their mutual relationship [40-44]. The dependency graph representation used in this work is "collapsed dependencies with propagation of conjunct dependencies" (section 4.3 of [45]). Compared to other representation styles provided by the Stanford parser, this representation approximates more closely the semantic relations in sentences by collapsing the dependencies involving prepositions, and propagating conjunction dependencies. Consequently, it helps to simplify the event rules for detecting Protein-Residue associations. However, this representation does not guarantee a tree structure, and may form a cyclic graph. Therefore, where there exists more than one shortest path between nodes, all of the paths are considered to avoid bias.

**Figure 4:**
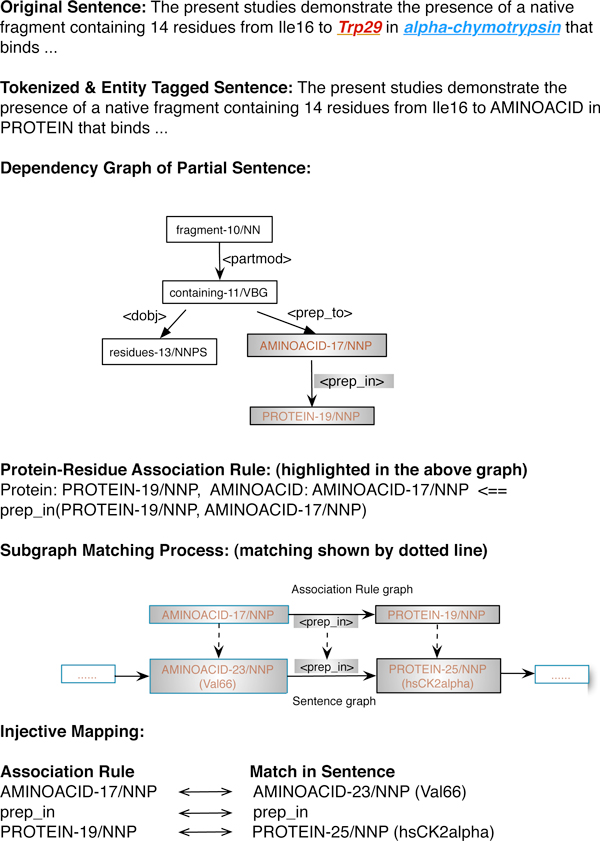
**Rule induction and protein-residue relation extraction**.

Figure 4 illustrates the rule induction process from an example sentence in the silver annotation. While the left hand side of the rule describes the protein-residue relation, the right hand side represents the dependency graph of the rule.

Subsequently we attempt to match the event rules to each test sentence to extract relevant events. Since the event rules and the sentences all possess a dependency graph, the matching process is casted as a subgraph matching problem [16], which corresponds to the search for a subgraph within the graph of a test sentence that is isomorphic to an event rule graph.

According to the GENIA corpus, on average there are about 24 words in one biomedical sentence, which correspond to the nodes in the dependency graph. Consequently, the input graphs of sentences and event rules are not large graphs. Therefore, we applied a simpler subgraph matching algorithm using a backtracking approach, developed by Liu et al. [16,17], to our matching process between rules and sentences.

When matching between graphs, different combinations of matching features can be applied, resulting in different matching criteria. The features include edge features (E) which are edge label and edge direction, and node features which are POS tags (P), and all tokens (A), ranging from the least specific matching criteria, E and P, to the much stricter criterion including A (E+P+A in Table 4). In addition, this subgraph matching algorithm inherently allows for the incorporation of existing knowledge such as ontological resources into the matching process between nodes or edges, to further improve the precision of the overall graph matching.

The algorithm proceeds until a subgraph isomorphic to the rule graph is found in the sentence graph. For each sentence, the algorithm returns all the matched rules together with the corresponding injective mappings from rule nodes to sentence tokens. Protein-residue relations are then extracted by identifying the specific protein and the amino acid/mutation in each sentence that correspond to the relationship specified in the matched rules.

Figure 4 also presents a simple example of the association extraction process by matching an event rule to a sentence. The matching criteria in the example require that edges be matched if they share a same direction and possess identical edge labels while nodes be matched if they belong to the same biological entity type.

The backtracking ability of the subgraph matching algorithm allows the relation extraction process to recover from initial wrong matches and continue to proceed until the correct protein-residue association is identified. In practice, it only takes the algorithm a couple of seconds to return the results. Hence, this algorithm is efficiently solving the subgraph matching problem in this work. More details and the complexity of the algorithm are presented in [16,17].

The graph-based approach used in this work has several advantages over some traditional machine learning methods. 1) It relies only on the positive instances and does not require negative examples for training. 2) Pattern induction through distant supervision where the relation between the protein and the residue mentions is known to exist in publicly available databases further reduces the risk of data over-fitting. 3) Unlike statistical methods, the approach produces a human-interpretable model, which allows more straightforward error analysis.

## Competing interests

The authors declare that they have no competing interests.

## Authors' contributions

KER and KV designed all the experiments described in this paper. KV designed the distant learning strategy. KER prepared the data sets, conducted the experiments and evaluated the performance of the system. KER also manually annotated the LEAP-FS corpus described in the paper. HL adapted his graph-based relation extraction system to the protein-residue association problem, provided his approximate subgraph matching algorithm, and assisted with performance analysis of those methods. JDC and MEW designed the physical validation algorithms and provided the data used to generate the silver corpus. KER, HL, and KV wrote the manuscript.
